# Fast Fight Detection

**DOI:** 10.1371/journal.pone.0120448

**Published:** 2015-04-10

**Authors:** Ismael Serrano Gracia, Oscar Deniz Suarez, Gloria Bueno Garcia, Tae-Kyun Kim

**Affiliations:** 1 Department of Systems Engineering and Automation, E.T.S.I. Industriales, Ciudad Real, Castilla-La Mancha, Spain; 2 Department of Electrical and Electronic Engineering, Imperial College, London, UK; Cajal Institute, Consejo Superior de Investigaciones Científicas, SPAIN

## Abstract

Action recognition has become a hot topic within computer vision. However, the action recognition community has focused mainly on relatively simple actions like clapping, walking, jogging, etc. The detection of specific events with direct practical use such as fights or in general aggressive behavior has been comparatively less studied. Such capability may be extremely useful in some video surveillance scenarios like prisons, psychiatric centers or even embedded in camera phones. As a consequence, there is growing interest in developing violence detection algorithms. Recent work considered the well-known Bag-of-Words framework for the specific problem of fight detection. Under this framework, spatio-temporal features are extracted from the video sequences and used for classification. Despite encouraging results in which high accuracy rates were achieved, the computational cost of extracting such features is prohibitive for practical applications. This work proposes a novel method to detect violence sequences. Features extracted from motion blobs are used to discriminate fight and non-fight sequences. Although the method is outperformed in accuracy by state of the art, it has a significantly faster computation time thus making it amenable for real-time applications.

## Introduction

In the last few years, the problem of human action recognition from video has become tractable by using computer vision techniques, see surveys [1], [2], [3]. Within this topic, there is a vast literature in which experimental results are given for recognition of human actions like walking, jumping or hand waving [4]. However, action detection has been devoted less effort. Action detection is a related task in which only a specific action must be detected. Action detection may be of direct use in real-life applications, fight detection being a clear example. Whereas there is a number of well-studied datasets for action recognition, significant datasets with violent actions (fights) have not been made available until the work [5]. A violence detector has, however, immediate applicability in the surveillance domain. The primary function of large-scale surveillance systems deployed in institutions such as schools, prisons and psychiatric care facilities is for alerting authorities to potentially dangerous situations. However, human operators are overwhelmed with the number of camera feeds and manual response times are slow, resulting in a strong demand for automated alert systems. Similarly, there is increasing demand for automated rating and tagging systems that can process the great quantities of video uploaded to websites.

One of the first proposals for violence recognition in video is Nam et al. [6], which proposed recognizing violent scenes in videos using flame and blood detection and capturing the degree of motion, as well as the characteristic sounds of violent events. Cheng et al. [7] recognize gunshots, explosions and car-braking in audio using a hierarchical approach based on Gaussian mixture models and Hidden Markov models (HMM). Giannakopoulos et al. [8] also propose a violence detector based on audio features. Clarin et al. [9] present a system that uses a Kohonen self-organizing map to detect skin and blood pixels in each frame and motion intensity analysis to detect violent actions involving blood. Zajdel et al. [10], introduced the CASSANDRA system, which employs motion features related to articulation in video and scream-like cues in audio to detect aggression in surveillance videos.

More recently, Gong et al. [11] propose a violence detector using low-level visual and auditory features and high-level audio effects identifying potential violent content in movies. Chen et al. [12] use binary local motion descriptors (spatio-temporal video cubes) and a bag-of-words approach to detect aggressive behaviors. Lin and Wang [13] describe a weakly-supervised audio violence classifier combined using co-training with a motion, explosion and blood video classifier to detect violent scenes in movies. Giannakopoulos et al. [14] present a method for violence detection in movies based on audio-visual information that uses a statistics of audio features and average motion and motion orientation variance features in video combined in a k-Nearest Neighbor classifier to decide whether the given sequence is violent. Chen et al. [15] proposed a method based on motion and detecting faces and nearby blood. Violence detection has been even approached using static images [16]. Also recently, [17] approached the problem within the context of video sharing sites by using textual tags along with audio and video. [18] recently approached the problem of detecting violence outbreaks in crowds using an optical flow-based method. Proof of the growing interest in the topic is also the MediaEval Affect Task, a competition that aims at discovering violence in color movies [19]. Goto et al. [20] recently proposed a system for violent scenes detection, which is based on the combination of visual and audio features at segment-level. In this case the algorithms have access to additional information such as audio, subtitles and previously-annotated concepts.

In summary, a number of previous works require audio cues for detecting violence or rely on color to detect cues such as blood. In this respect, we note that there are important applications, particularly in surveillance, where audio and color are not available. In other cases it is possible and easy to obtain audio, but audio features can increase false positive rates or miss true positives because there are many violence situations where the audio signal is not significant, for example: to push, throw (something), knock down, attack with a knife, block (someone), etc. Besides, while explosions, blood and running may be useful cues for violence in action movies, they are rare in real-world situations. In any case, violence detection is an extremely difficult problem, since violence is a subjective concept. Fight detection, on the contrary, is a specific violence-related task that may be tackled using action recognition techniques.

Deniz et al. [21] have more recently presented a novel method to detect violent sequences which uses extreme acceleration patterns as the main feature. The average performance was around 90% in three datasets and with a feature extraction time of 162 ms (including a necessary global motion correction step). Bermejo et al. [5] recently demonstrated encouraging results in applying generic action recognition methods to violent detection, achieving 90% accuracy using MoSIFT features ([22]). MoSIFT descriptors are obtained from salient points in two parts: the first is an aggregated histogram of gradients (HoG) which describe the spatial appearance. The second part is an aggregated histogram of optical flow (HoF) which indicates the movement of the feature point. MoSIFT are powerful features used for generic action recognition. However, the computational cost of extracting such features is prohibitively large, taking nearly 1 second per frame on a high-end laptop. This precludes use in practical applications, where many camera streams may have to be processed in real-time. Such cost is also a major problem when the objective is to embed a fight detection functionality into a smart camera (i.e. going from the extant embedded motion detection to embedded violent motion detection). Also these cameras could provide global motion compensation that can be performed in the device itself.

In this context, an efficient method is proposed based on detecting motion blobs. Afterwards, features extracted from these blobs are ultimately used to discriminate between fight and non-fight sequences. The paper is organized as follows. Section describes the proposed method. Section provides experimental results. Finally, in Section the main conclusions are outlined.

## Method

**Fig 1:**
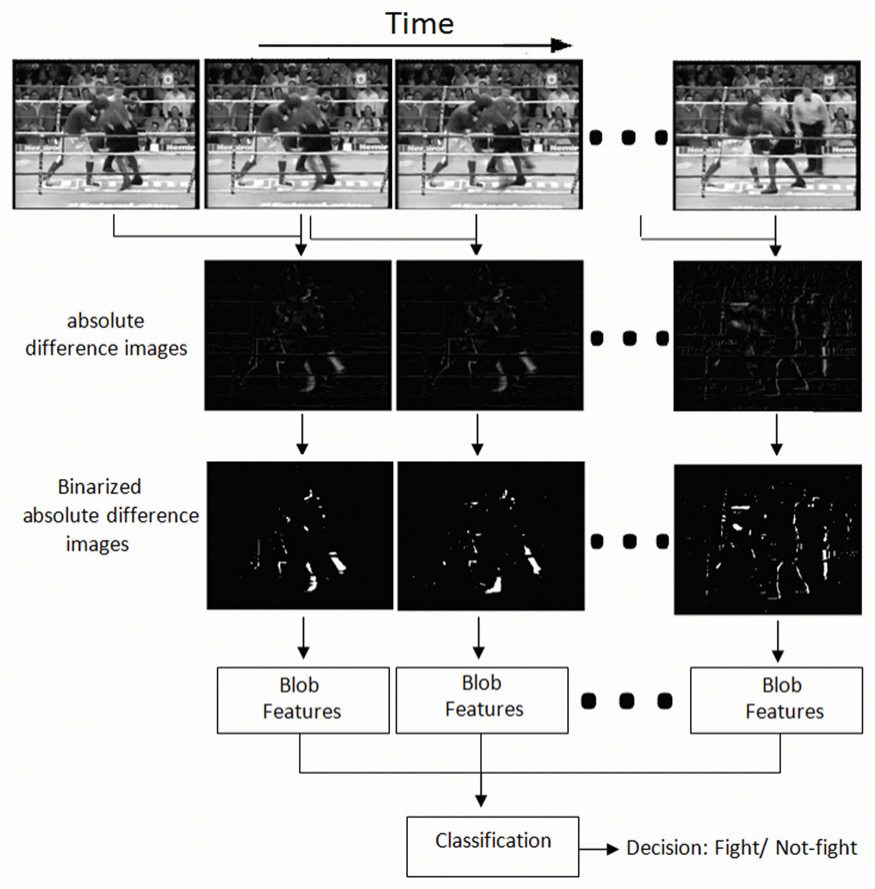
General diagram. General diagram of the proposed method.

**Fig 2:**
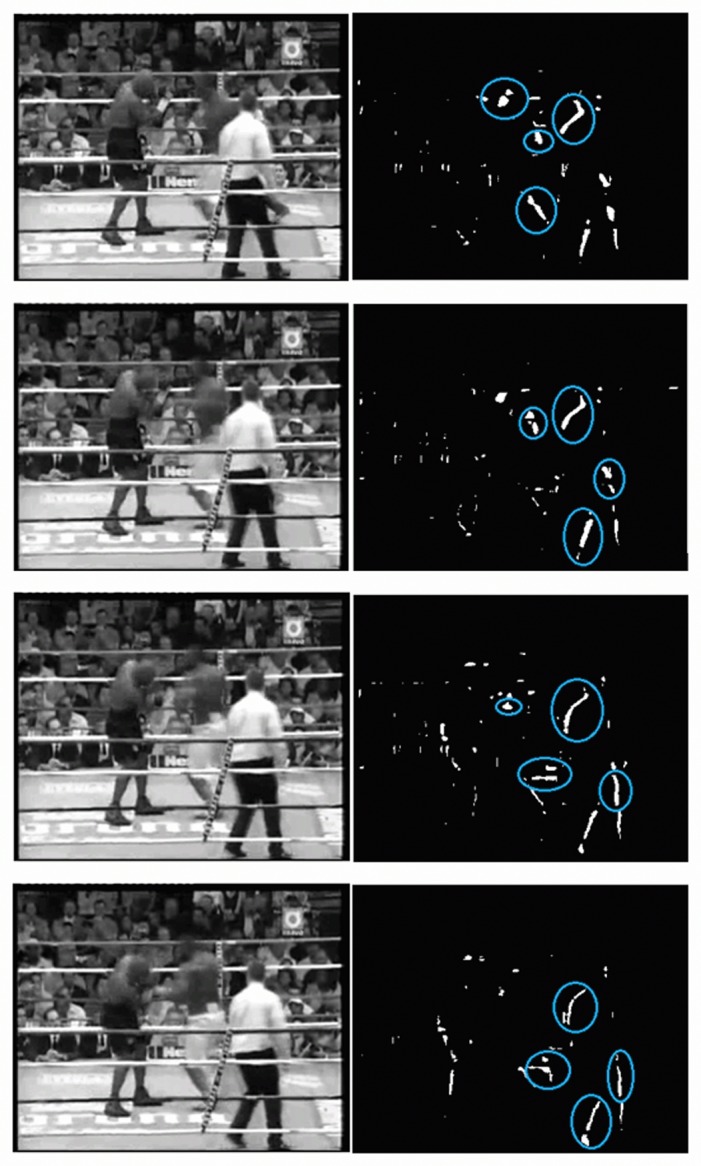
Fight sequence. Four consecutive frames from a fight sequence where the four largest motion blobs have been marked.

**Fig 3:**
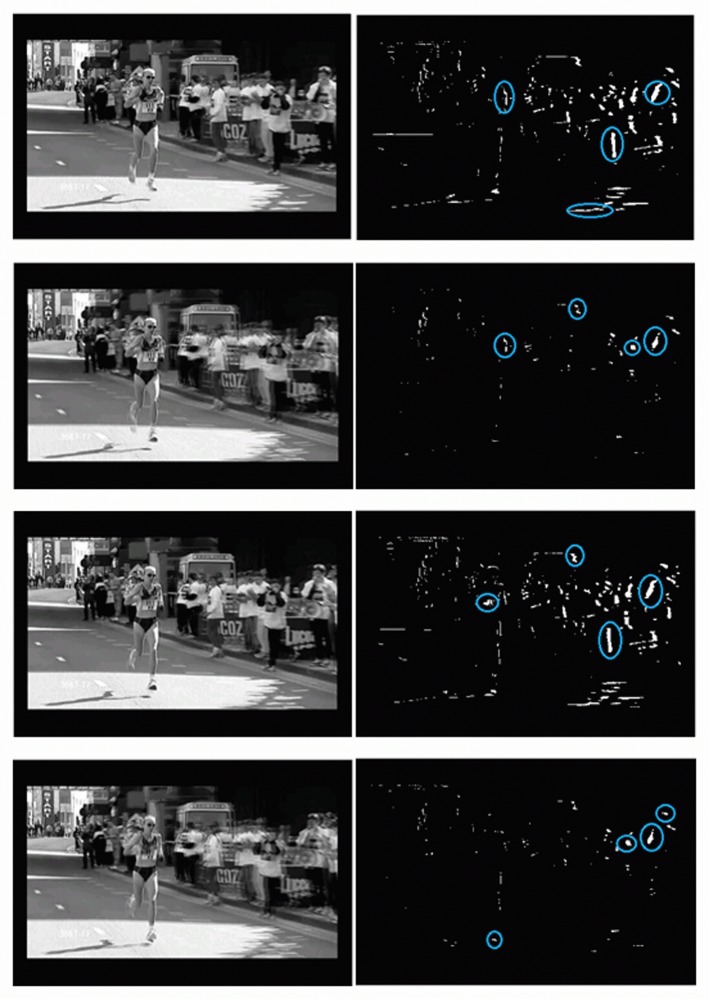
Non-Fight sequence. Four consecutive frames from of a non-fight sequence where the four largest motion blobs have been marked. Compare with those of Fig 2. In the previous figure the motion blobs are larger and clustered, whereas in the current figure the motion blobs are smaller and not clustered.

As mentioned above, the main steps of the proposed method are shown in Fig 1. It is hypothesized that motion blobs in fight sequences have a distinct position and shape. Firstly, the absolute image difference between consecutive frames is computed. The resulting image is then binarized, leading to a number of motion blobs, see Fig 2 where the largest blobs have been marked on a fight sequence and Fig 3 on a no-fight sequence. Only the *K* largest motion blobs are selected for further processing.

In order to characterize the *K* blobs, different measures are computed such as area, centroid, perimeter, … as well as distances between blob centroids. In the following, the method is described in detail.

A short sequence *S*(*s*) of gray scale images is denoted as:

$$\begin{array}{c} S\left(s\right)=I_{t}\left(x,y\right) \end{array}$$

where *x* = 1, 2, …, *N*, *y* = 1, 2, …, *M* and *t* = 1, 2, …, *T*. *N* and *M* are the number of rows and columns of each frame *I* and *T* is the number of frames respectively.

Let *I*_*t*−1_(*x*, *y*) and *I*_*t*_(*x*, *y*) be two consecutive frames in the sequence. The absolute difference between consecutive images is then computed as:

$$\begin{array}{c} E_{t}\left(x,y\right)=\left|I_{t-1}\left(x,y\right)-I_{t}\left(x,y\right)\right| \end{array}$$

Then, *E* is binarized using a threshold *h*:

$$\begin{array}{c} F_{t}\left(x,y\right)=\left\{\begin{array}{cc} 1, & \text{if}\;E_{t}\left(x,y\right)>255*h\text{,}\\ 0, & \text{otherwise} \end{array}\right. \end{array}$$

where 0 < *h* < 1 can be arbitrarily chosen. The second step is to define each blob on the image *F*_*i*_(*x*, *y*). Each blob (*B*) is the set of pixels (*x*, *y*) for which *B*_*b*,*t*_(*x*, *y*) = *b*, where *b* = 0, 1, 2, …, *J* and where *J* is the number of blobs in image *F*_*t*_(*x*, *y*). Each blob *B*_*b*,*t*_(*x*, *y*) is here defined as an image containing a set of adjacent points, neighborhood (𝒩), where *F*_*t*_(*x*, *y*) = 1.

$$\begin{array}{c} B_{b,t}\left(x,y\right)=\{\begin{array}{cc} 1, & \text{if}\;\underset{\left(x,y\right)\in {𝒩}_{1}}{\cup }F_{t}\left(x,y\right)=1\text{,}\;\sum^{}(\left(x,y\right)\in {𝒩}_{1})=m_{1}\text{,}\;\\ 2, & \text{if}\;\underset{\left(x,y\right)\in {𝒩}_{2}}{\cup }F_{t}\left(x,y\right)=1\text{,}\;\sum^{}(\left(x,y\right)\in {𝒩}_{2})=m_{2}\text{,}\;\\ \ldots & \\ J, & \text{if}\;\underset{\left(x,y\right)\in {𝒩}_{J}}{\cup }F_{t}\left(x,y\right)=1\text{,}\;\sum^{}(\left(x,y\right)\in {𝒩}_{J})=m_{J}\text{,}\;\\ 0, & \text{otherwise} \end{array} \end{array}$$

where *m*_1_, *m*_2_, … *m*_*J*_ are the number of adjacent pixels of each blob respectively and *m*_1_+*m*_2_+…*m*_*J*_ ≤ *N* ⋅ *M*. These blobs are calculated for every frame. Afterwards, the *K* largest blobs are selected to extract useful information for the classification of the video sequences. The following features are extracted by the proposed method.

The blob areas (*A*_*a*,*t*_) will be defined**l**ike as:

$$\begin{array}{c} A_{a,t}=\frac{\sum_{x=1}^{N}\sum_{y=1}^{M}B_{a,t}\left(x,y\right)}{a} \end{array}$$

where *a* = 1, 2, …, *K*. The centroids (*CX*_*c*,*t*_ and *CY*_*c*,*t*_) are

$$\begin{array}{c} CX_{c,t}=\frac{\sum_{x=1}^{N}B_{c,t}\left(x,y\right)*x}{\sum_{x=1}^{N}B_{c,t}\left(x,y\right)}=\frac{\sum_{x=1}^{N}B_{c,t}\left(x,y\right)*x}{N} \end{array}$$

$$\begin{array}{c} CY_{c,t}=\frac{\sum_{y=1}^{M}B_{c,t}\left(x,y\right)*y}{\sum_{y=1}^{M}B_{c,t}\left(x,y\right)}=\frac{\sum_{y=1}^{M}B_{c,t}\left(x,y\right)*y}{M} \end{array}$$

where *c* = 1, 2, …, *K*. The best *K* blobs are selected according to area (largest *K* areas). Now, the distances *D*_*d*,*t*_ between blobs are calculated as:

$$\begin{array}{c} D_{d,t}=\sqrt{{\left(CX_{d+1,t}-CX_{d,t}\right)}^{2}+{\left(CY_{d+1,t}-CY_{d,t}\right)}^{2}} \end{array}$$

where $d=1,2,\;\ldots ,\sum_{i=1}^{K-1}i$. Therefore, *D*_*d*,*t*_ represents the distances between every pair in the *K* blobs.

Compactness (*C*_*co*,*t*_) is also used to estimate the shape (rounded or elliptical) of the blob and is defined as:

$$\begin{array}{c} C_{co,t}=\frac{P_{co,t}^{2}}{A_{co,t}} \end{array}$$

where *co* = 1, 2, …, *K*. To estimate the perimeter (*P*_*p*,*t*_) the Sobel operator [23] has been used to detect the edges over *B*_*b*,*t*_(*x*, *y*). This operator (*G*_*b*,*t*_(*x*, *y*)) is applied to calculate the edges for each blob. Edge pixels have value 1 and the rest 0.

$$\begin{array}{c} P_{p,t}=\frac{\sum_{x=1}^{N}\sum_{y=1}^{M}G_{p,t}\left(x,y\right)}{p} \end{array}$$

where *p* = 1, 2, …, *K*.

**Fig 4:**
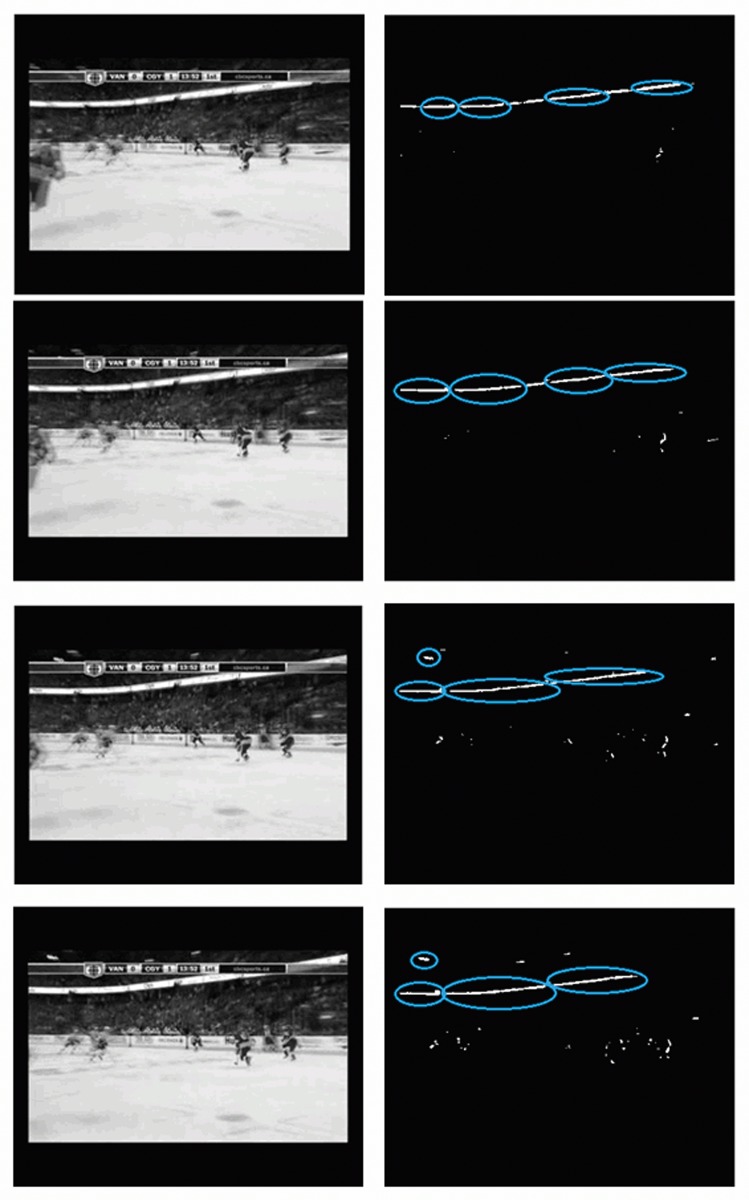
Global movement sequence. Four consecutive frames from with global movement where the four largest motion blobs have been marked.

Finally, note that the proposed method is intrinsically able to learn in the presence of global motion. Global motion leads to blobs with a distinct shape and position, typically large elongated blobs, see Fig 4.

## Results

### Datasets

**Fig 5:**
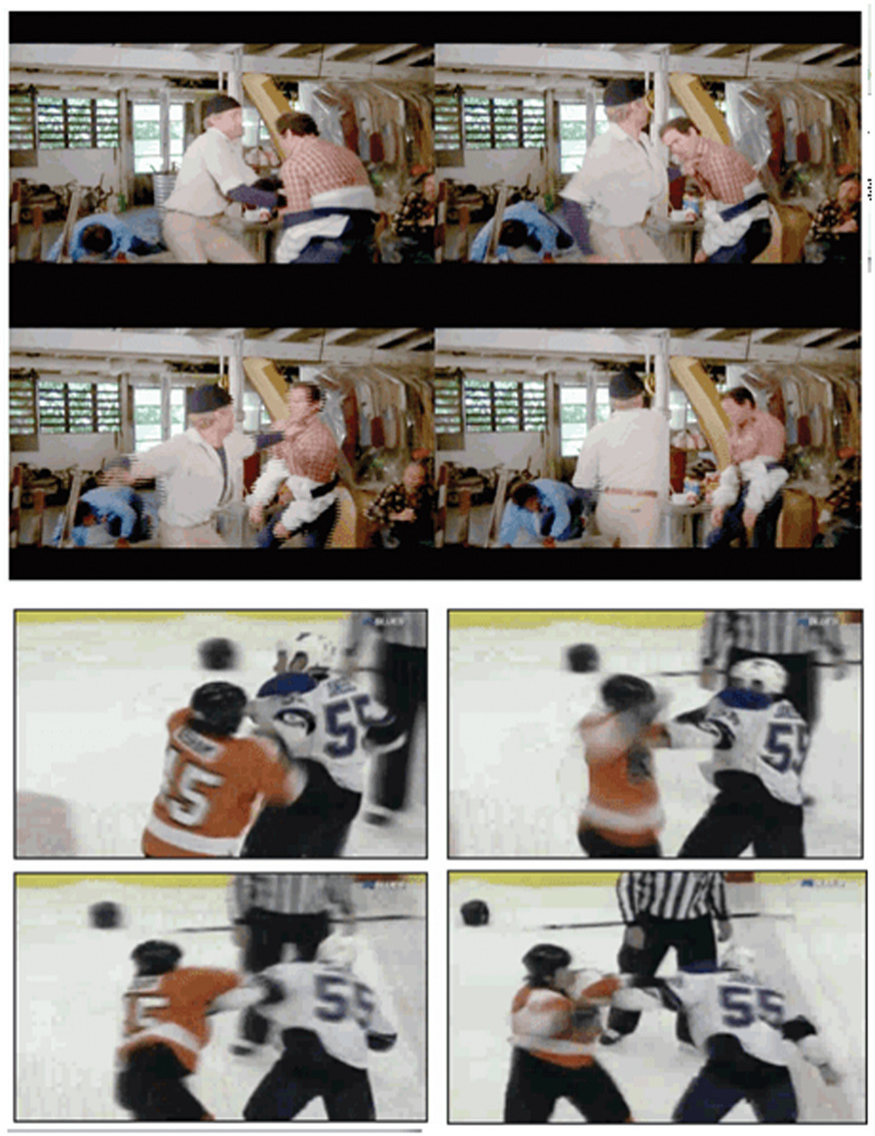
Movie and Hockey datasets. Sample fight videos from the action movie (top) dataset and the Hockey (bottom) datase

The proposed method was assessed using three different datasets and compared with other five related methods. The work [5] introduced the first two datasets explicitly designed for assessing fight detection. The first dataset (“Movies”) introduced in [5] consists of 200 video clips in which fights were extracted from action movies (see Fig 5 top). The non-fight videos were extracted from public action recognition datasets. The second dataset (“Hockey”) consists of 1000 clips at a resolution of 720×576 pixels, divided in two groups, 500 fights (see Fig 5 bottom) and 500 non-fights, extracted from hockey games of the National Hockey League (NHL). Each clip was limited to 50 frames and resolution lowered to 320x240. Unlike the Hockey dataset, which was relatively uniform both in format and content, the movies dataset had a wider variety of scenes that were captured at different resolutions. This dataset was rescaled to a uniform size.

**Fig 6:**
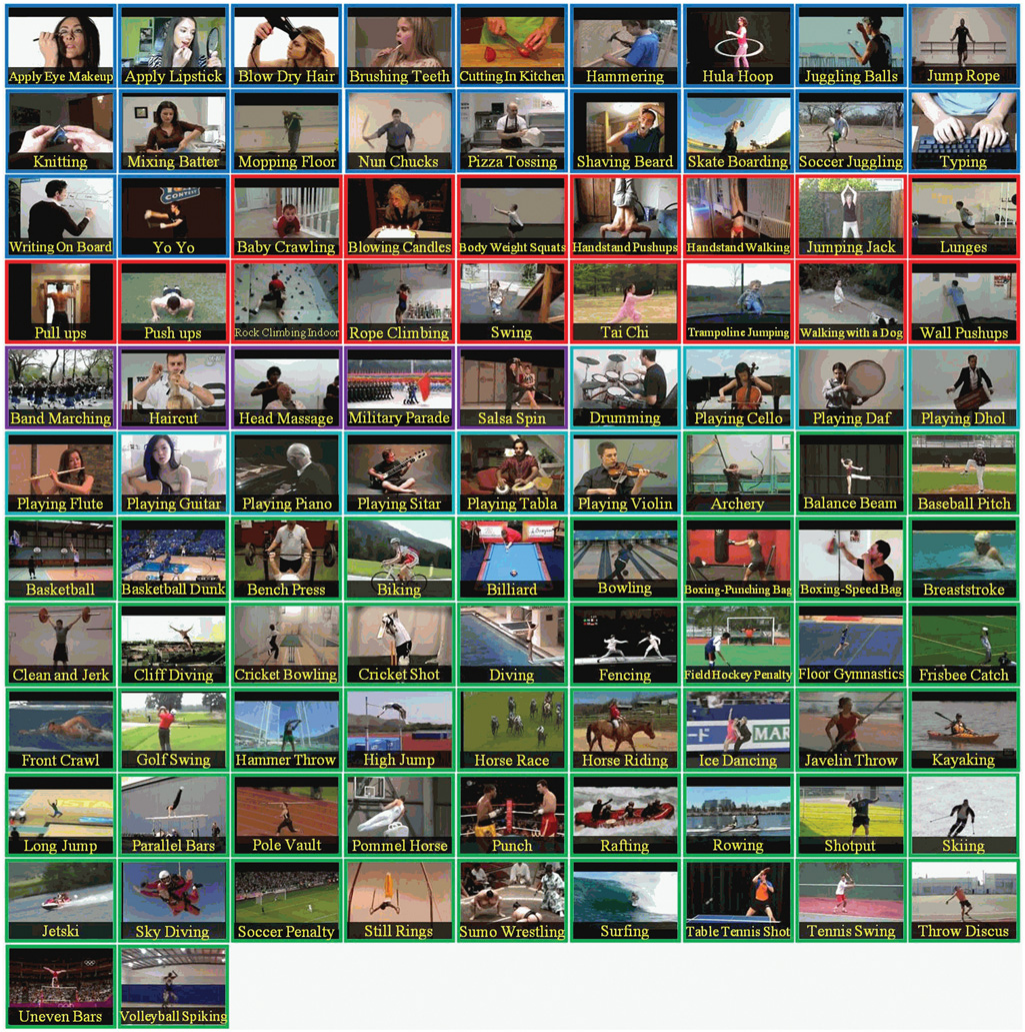
UCF101 dataset. The 101 actions in UCF101 shown with one sample frame.

Since the proposed method is based to detect blobs, sudden actions such as those in the above-mentioned Hockey dataset are particularly challenging. In any case, for practical use the aim at separating fights from other typical actions. Consequently, a more realistic dataset was also considered. The UCF101 [24] is a data set of realistic action videos collected from YouTube, having 101 action categories. UCF101, see Fig 6, gives the largest diversity in terms of actions and with the presence of large variations in camera motion, object appearance and pose, object scale, viewpoint, cluttered background and illumination conditions it is the most challenging dataset to date. For this case, it is even more challenging since it also includes 50 actions from sports. To our knowledge, this is the largest and most challenging dataset in which a fight detection algorithm has been tested.

**Fig 7:**
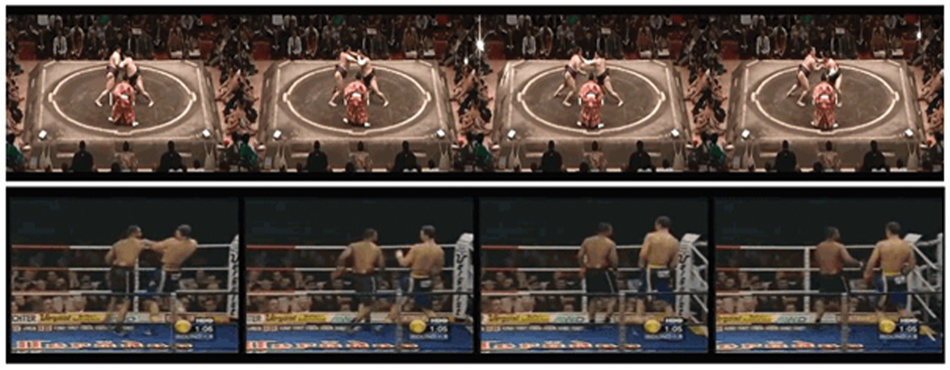
Fights on UCF101. Sample frames of Sumo and Punch categories in UCF101.

In the experiments with UCF101, for the fight set was pooled on the fight clips of both the Hockey and Movies dataset plus two of the 101 UCF actions that actually represented fights (“Punching” and “Sumo”, see Fig 7). This gave a total of 1843 fight clips and 42278 non-fight clips (totaling approximately 2 Million frames). In order to avoid unbalanced sets have been selected a randomly chosen subset of 500 fight and 500 non-fight clips. This in turn was repeated 10 times.

### Experimental Setup

In the following experiments, two variants of the proposed method are described, called **v1** and **v2**. Previously some of the related methods are commented.

As for the other related methods, the Bag-of-Words framework was used with both STIP and MoSIFT features, as in [5]. In [5] STIP features performed poorly on the Movie dataset and so MoSIFT was considered the best descriptor. MoSIFT’s superiority has been also proven in other action recognition works. For BoW(MoSIFT), and even with the use of parallel K-means, extracting vocabularies from the whole UCF101 dataset was unfeasible. Therefore, a random subset of 1200 samples was first selected and then a vocabulary of size 500 (the best vocabulary size in [5]) was computed (The authors are not aware of any previous work using MoSIFT in a bag-of-words framework with the UCF101 database).

The Violent Flows method (ViF) [18] mentioned in Section was also tested. ViF is a recent method which may be considered representative of dense optical flow based methods for action recognition (other examples that fall into this category are the dense trajectories of [25] and discriminate slow feature analysis [26]). Finally, results are also compared to the Local Motion Patterns method (LMP) [27]. This method is based on extracting simple statistics (variance, skewness and kurtosis) from temporal cuboids centered on tracked keypoints. Keypoints are located with a Harris detector. In [27] the authors claimed that this descriptor is both informative and efficient for action recognition. Since the number of extracted descriptor vectors varies in each video sequence is extracted fixed-size histograms for each feature, similar to what is done with the proposed method. The best results were obtained using 6 bins. Deniz et al. [21] method is another recent method to detect violent sequences mentioned in Section and was also included in the experiments.

The first variant (**v1**) assumes that position and shape of *K* largest blobs can discriminate between fight and non-fight sequences. To characterize position and shape, the centroid and distances between blob centroids are computed. In summary, this variant (**v1**) uses the best *K* areas, the *K* centroids of these areas and the distances between these centroids. Hence, a sequence *S* has 3*K* + *K*(*K* − 1)/2 features.

The second variant (**v2**) uses the following features to characterize position and shape, the centroid, distances between blob centroids and compactnesses. Finally, this variant (**v2**) uses the best *K* areas, the *K* centroids of these areas, the distances between these centroids and the *K* compactnesses. Hence, the sequence *S* has 2*K* + *K*(*K* − 1)/2 features.

In the experiments, the proposed method needs to set two parameters. First, a binary thresholding parameter *h* between 0 < *h* < 1 to binarize this image. Second, the parameter *K* that represents the number of largest blobs (Areas, Centroids, distances between centroids and compactnesses, see the previous Section). The Movies dataset has been used to fit these parameters. To find the best configuration of the proposed method Accuracy has been used. The Accuracy is the number the True Posives(TP) plus True Negatives(TN) divided by the total number of samples. Tables 1 and 2 show the results measured using 5 repetitions of 10-fold cross-validation and three different classifiers KNN, Adaboost (100 classifiers, weighted voting) and Random Forests (50 trees) for both variants (**v1** and **v2**) of the method. The aim is to set the best parameters *h* and *K* for these two variants. These tables (1 and 2) show the results. The *h* and *K* parameters have been set to 0.4 and 8 respectively for both variants. In the following, these two variants are compared with other extant methods.

**Table 1:** This table shows the mean and standard deviation of the Accuracy for these three classifiers for variant 1 of the proposed method.

Method	*h*	*K*	KNN	AdaBoost	Random Forests
Variant-v1	0.2	4	91.1 ± 0.7	79.5 ± 0.6	95.9 ± 0.5
		6	92.6 ± 0.5	84.9 ± 0.8	94 ± 0.6
		8	95 ± 0.4	87.7 ± 1.1	**96.4 ± 0.5**
		10	93.5 ± 0.2	89.2 ± 0.4	95.5 ± 0.4
		12	94.7 ± 0.4	90.6 ± 0.6	95.2 ± 0.5
		14	93.5 ± 0.2	90.2 ± 0.6	95.0 ± 0.6
		16	93.3 ± 0.8	89.4 ± 0.4	95.1 ± 0.5
		18	93 ± 0.4	88.1 ± 0.6	95.2 ± 0.8
		20	92.8 ± 0.4	87.2 ± 0.3	94.8 ± 0.4
	0.05	8	88.6 ± 0.1	78.5 ± 0.7	93.8 ± 0.3
	0.1		90.2 ± 1	78.4 ± 0.8	94.4 ± 0.7
	0.15		88.5 ± 0.4	83.6 ± 0.5	96.1 ± 0.3
	0.25		93.6 ± 0.7	88 ± 0.9	96.1 ± 0.3
	0.3		94.8 ± 0.4	87.8 ± 0.6	97.4 ± 0.4
	0.35		90.6 ± 1.9	85.1 ± 0.4	97.1 ± 0.3
	0.4		63.4 ± 3.8	81.8 ± 0.2	**97.7 ± 0.4**
	0.45		91.7 ± 1.1	79.6 ± 0.8	94.8 ± 0.4
	0.5		94 ± 0.2	77.7 ± 0.3	92.7 ± 0.4

**Table 2:** This table shows the mean and standard deviation of the Accuracy for three classifiers for variant 2 of the proposed method.

Method	*h*	*K*	KNN	AdaBoost	Random Forests
Variant-v2	0.2	4	92.7 ± 0.8	79.3 ± 0.8	95.9 ± 0.3
		6	93.2 ± 0.6	84.5 ± 1.5	95.7 ± 0.6
		8	95.2 ± 0.8	88.6 ± 0.6	**96.4 ± 0.3**
		10	93.7 ± 0.4	90.2 ± 0.4	95 ± 0.8
		12	94.5 ± 0.5	90.6 ± 0.7	95.9 ± 0.9
		14	93.9 ± 0.5	90.7 ± 0.4	95.1 ± 0.9
		16	94 ± 0.4	90 ± 0.2	95.5 ± 0.8
		18	93.7 ± 0.3	88.2 ± 0.5	95.2 ± 1
		20	92.6 ± 0.5	87.2 ± 0.3	94.4 ± 0.5
	0.05	8	88.8 ± 1.2	77.3 ± 0.4	93.9 ± 0.5
	0.1		91.2 ± 0.6	78.8 ± 1.4	94.9 ± 0.7
	0.15		89.7 ± 1.3	83.1 ± 1.4	96.1 ± 0.5
	0.25		93 ± 0.4	88.4 ± 1.2	95.8 ± 0.6
	0.3		94.8 ± 0.4	88.4 ± 0.6	97.3 ± 0.2
	0.35		88.2 ± 2	85.3 ± 0.6	97.4 ± 0.2
	0.4		65.1 ± 2.7	81.8 ± 0.4	**97.8 ± 0.4**
	0.45		91.2 ± 2.3	79.5 ± 0.5	94.9 ± 0.5
	0.5		91 ± 4.3	77.2 ± 0.3	92.6 ± 0.3

### Discussion

**Fig 8:**
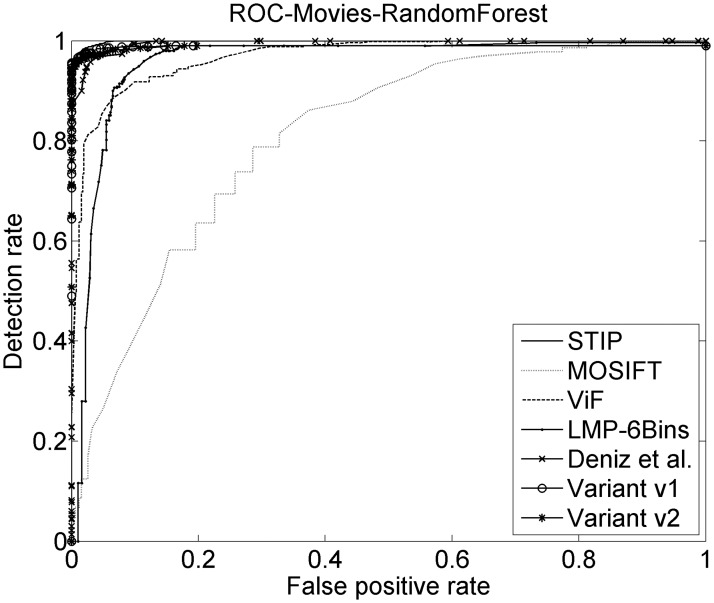
ROCs on Movies dataset. ROC curves for the five related methods and the two considered variants. The Random Forests classifier on Movies dataset is used.

**Fig 9:**
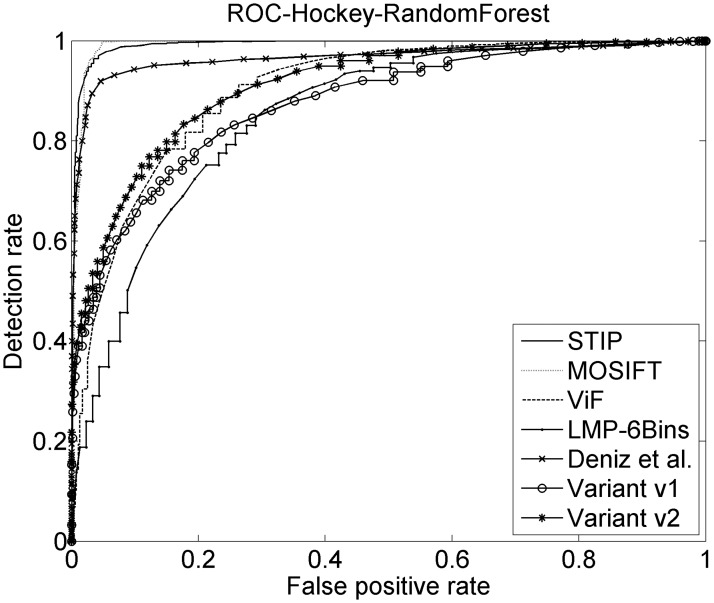
ROCs on Hockey dataset. ROC curves for the five related methods and the two considered variants. The Random Forests classifier on Hockey dataset is used.

**Fig 10:**
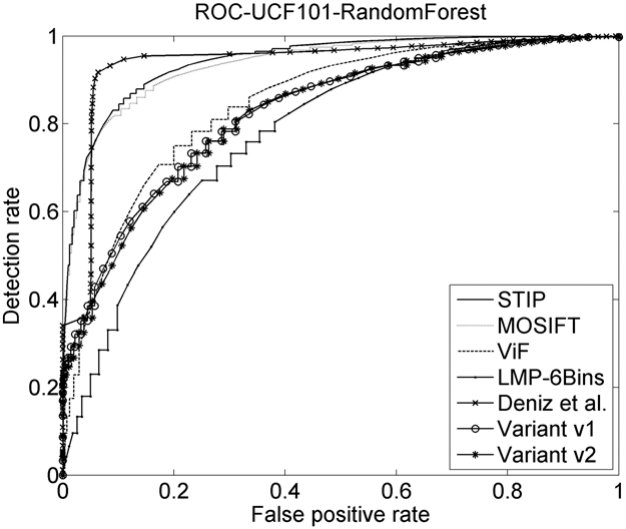
ROCs on UCF101 dataset. ROC curves for the five related methods and the two considered variants. The Random Forests classifier on UCF101 dataset is used.

The related methods are now compared with the two variants proposed. See Table 3 which shows the results for the three datasets using Support Vector Machines (SVM), AdaBoost and Random Forests (RF) classifiers are compared. See Figs 8, 9 and 10 where the ROC can be observed for the Random Forest classifier for each method.

**Table 3:** This table shows the results for the three datasets using Support Vector Machines (SVM), AdaBoost and Random Forests (RF) classifiers for the related methods and the two proposed variants. Three measures have been calculated: mean accuracy, standard deviation accuracy and AUC.

Method	Classifier	Dataset		
				Movies	Hockey	UCF101
BoW (STIP)	SVM	82.3±0.9/0.88	88.5±0.2/0.95	72.5±1.5/0.74
	AdaBoost	75.3±0.83/0.83	87.1±0.2/0.93	63.1±1.9/0.68
	RF	97.7±0.5/0.99	96.5±0.2/0.99	87.3±0.8/0.94
BoW (MoSIFT)	SVM	63.4±1.6/0.72	83.9±0.6/0.93	81.3± 1/0.86
	AdaBoost	65.3±2.1/0.72	86.9±1.6/0.96	52.8±3.6/0.62
	RF	75.1±1.6/0.81	**96.7±0.7/0.99**	86.3±0.8/0.93
ViF	SVM	96.7±0.3/0.98	82.3±0.2/0.91	77.7±2.16/0.87
	AdaBoost	92.8±0.4/0.97	82.2±0.4/0.91	78.4±1.7/0.86
	RF	88.9±1.2/0.97	82.4±0.6/0.9	77±1.2/0.85
LMP	SVM	84.4±0.8/0.92	75.9±0.3/0.84	65.9±1.5/0.74
	AdaBoost	81.5±2.1/0.86	76.5±0.9/0.82	67.1±1/0.71
	RF	92±1/0.96	77.7±0.6/0.85	71.4±1.6/0.78
Deniz et al. [21]	SVM	85.4±9.3/0.74	90.1±0/0.95	**93.4±6.1/0.94**
	AdaBoost	**98.9±0.22/0.99**	90.1±0/0.90	92.8±6.2/0.94
	RF	90.4±3.1/0.99	61.5±6.8/0.96	64.8±15.9/0.93
Variant-v1	SVM	87.9±1/0.97	70.8±0.4/0.75	72.1±0.9/0.78
	AdaBoost	81.8±0.5/0.82	70.7±0.2/0.7	71.7±0.9/0.72
	RF	97.7±0.4/0.98	79.3±0.5/0.88	74.8±1.5/0.83
Variant-v2	SVM	87.2±0.7/0.97	72.5±0.5/0.76	71,2±0.7/0.78
	AdaBoost	81.7±0.2/0.82	71.7±0.3/0.72	71±0.8/0.72
	RF	97.8±0.4/0.97	82.4±0.6/0.9	79.5±0.9/0.85

**Fig 11:**
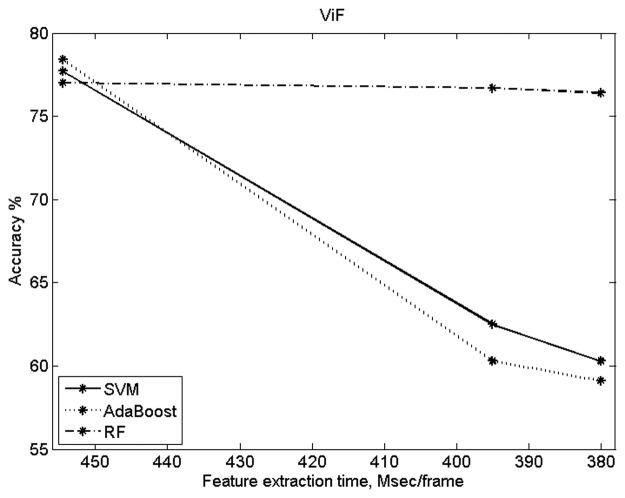
ViF results comparing accuracy vs feature extraction time.

**Fig 12:**
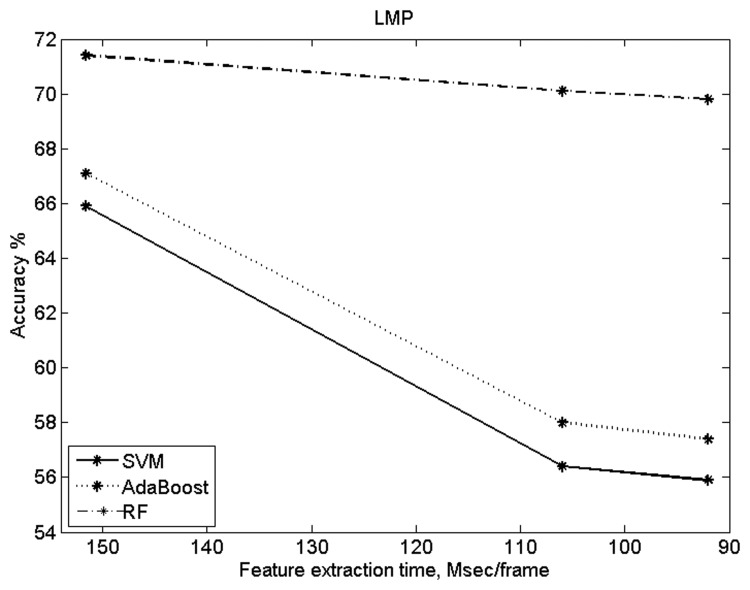
LMP results comparing accuracy vs feature extraction time.

In the experiments above, the ViF and LMP implementations followed the original version in the respective papers. In order to evaluate the dependence of accuracy of these methods on the number of features, it was also deemed necessary to assess the accuracy vs speed trade-off offered by those two methods. Fig 11 shows the results obtained with ViF (using UCF101 dataset). In this case the ViF results were obtained by varying the width of the coarsest level of the Optical Flow computation. As for LMP, the results (see Fig 12) were obtained by varying the number of temporal scales considered by the algorithm.

Table 4 shows the number of features used for classification and the computational cost (for feature extraction). The code for both STIP and MoSIFT was compiled, while the code for rest of the methods was interpreted and used no parallelization. These results show a significant improvement in speed. The proposed method, using 2548 and 2940 features respectively, is computationally 12 and 16 times faster than the STIP and MoSIFT methods.

**Table 4:** Feature extraction times. Average times measured with the non-fight videos in the UCF101 dataset, on an Intel Xeon computer with 2 processors at 2.90Ghz.

Method	Features/sequence	Msecs/frame
BoW(MoSIFT)	500	661.5
BoW(STIP)	500	293.5
ViF	96000	454.5
LMP	10368	151.6
Deniz et al. [21]	14	162.4
Variant-v1	2548	22.5
Variant-v2	2940	26.5

From the results it can be observed that the proposed method is not better than BoW(STIP), BoW(MoSIFT) and Deniz et al. [21] in terms of accuracy. However, the proposed method gives slightly better accuracies than the ViF and LMP methods. Most importantly, see Table 4, the proposed method is approximately 26, 12, 18, 6 and 7 times faster than the BoW(STIP), BoW(MoSIFT), ViF, LMP and Deniz et al. [21] methods respectively.

The proposed method is amenable to real-time applications since it requires less than 1/25 seconds per frame. In fact, the time spent in deciding whether an input sequence (50 frames) contains a violent action is between 0.0225 and 0.0265 seconds per frame.

The novel method has a processing average time of 0.02391 seconds per frame (between **v1** and **v2**). The method has different sub-steps for each frame. Some of these sub-steps can be parallelized when the image can be splitted in different smaller sub-images without affecting the result. Each sub-image can be handled by a processor. These sub-steps are shown in Table 5.

**Table 5:** Sub-steps of the proposed method.

Sub-step	Seconds per frame	Parallelizable?
Load a frame	0.00608	No
Convert RGB to Gray image	0.00412	Yes
Rescale	0.00749	Yes
Binarization	0.00041	Yes
Find the regions	0.00551	No
Select the K best regions	0.00022	No
Calculate the features	0.00008	Yes

Given a computer with N processors and parallelizable processes, the processing time can be theoretically estimated from Table 5 as *T* = *parallelizable*_*processes*/*N* + *not*_*parallelizable*_*processes*. For example, using a computer with 4 processors, the processing time would be *T* = 0.0121/4 + 0.01181, *T* = 0.01483 seconds per frame. This is an increase of 37.95% on the processing time.

## Conclusions

This work has described a novel method for detecting fights. Blobs of movement are first detected and then different features are used to characterize them. The proposed method makes no assumptions on number of individuals (it can be also used to detect vandalism), body part detection or salient point tracking. Experiments show that the method does not outperform the best methods considered. However, it is much faster while still maintaining useful accuracies ranging from 70% to near 98% depending on the dataset. While other methods tackle a more general problem (such as action recognition) or resort to computationally intensive optical flow computations, the proposed method opens up the possibility of practical implementations. There is growing interest in the private video surveillance sector in deploying efficient methods for violence detection in prisons and other premises.

The shortcomings of the proposed method are the following. The classification accuracy is not as good as the best state-of-art-the fight detection methods. Furthermore, the proposed method has difficulty in classifying videos where there is continuous movement from such as with clouds or tree branches when they are moved by the wind.

Future work will seek to improve accuracy by using additional features to detect the violent areas. Further speed improvements can be also obtained by exploiting parallelism in blob processing.
